# Editorial: Immunology of the Oral Mucosa

**DOI:** 10.3389/fimmu.2022.877209

**Published:** 2022-03-25

**Authors:** Lesley Ann Bergmeier, Nicolas Dutzan, Patricio C. Smith, Heleen Kraan

**Affiliations:** ^1^ Centre for Immunobiology and Regenerative Medicine, Barts and The London School of Medicine and Dentistry, Queen Mary University of London, London, United Kingdom; ^2^ Department of Conservative Dentistry, Faculty of Dentistry, University of Chile, Santiago, Chile; ^3^ School of Dentistry, Faculty of Medicine, Pontificia Universidad Católica de Chile, Santiago, Chile; ^4^ Institute for Translational Vaccinology, Intravacc, Bilthoven, Netherlands

**Keywords:** oral mucosa, biomarkers, Tregs (regulatory T cells), innate phagocytes, dysbiosis

The oral mucosa is host to over 700 species of commensal organisms (HTTP://www.homd.org) and is constantly exposed to potentially inflammatory stimuli, yet, in healthy individuals, acute inflammation is unusual. Waldeyer’s ring and numerous draining lymph nodes provide inductive and effector sites for intense immune activity that maintains the integrity of the mucosal barrier. A state of immune privilege or tolerance is said to exist in the oral mucosa through the induction of T regulatory cells and the production of cytokines that support immuno-suppression of unwanted responses to innocuous antigens (1–3). Several different types of stratified epithelia are present including the lining mucosa, masticatory and specialized mucosa and range from thin non-keratinized sub-lingual and buccal mucosae to thick highly keratinized Gingiva (4) Their functions have direct effects on immune responses, such as the induction of homeostatic Th17 responses by the mechanical damage of mastication (5).

Investigations of the oral cavity range from the first observations of dental plaque by Anton van Leeuwenhoek in the 1660’s to the recently published cell atlas of the oral mucosa (6) and have provided major contributions to the understanding of mucosal immune responses (7). Seminal studies emphasizing the importance of the ecological balance of the oral microbiota and the host have described dysbiosis as a driving force contributing to oral (and systemic) diseases (8).

This Research Topic has attracted an eclectic series of reviews, original research, case studies and hypotheses which are reflective of the importance and broad range of oral immunology studies and the applicability of the oral mucosa for diagnosis, intervention and prevention of disease.

Suárez et al. have compared the immune niches of the oral and gastrointestinal mucosa in terms of their anatomy, cell to cell communication, antigen handling, signaling pathways and systemic consequences in disease pathogenesis. A greater diversity of dendritic cells in the oral cavity (9), and potential synergistic interaction between TLRs and NOD receptors results in a measured response to oral bacteria (10). TLR splice variants have been observed in the buccal mucosa of the autoinflammatory condition Behçet’s Disease (11) where dysregulated antigen recognition could contribute to inflammatory profiles. Suárez et al. conclude that microbial translocation contributes to systemic disease and emphasize the bidirectionality of the interface between the oral and gastrointestinal mucosa.

An intriguing paper on obstructive sleep apnea by Samiento Varón et al. suggests that children with hypertrophied tonsils have abnormally active pro-inflammatory B and T cells, where modulation of the microbiome allows penetration into tonsil tissues and potential breaching of the epithelial barrier and is supported by previous studies (12).

Characterization of the human oral mucosa cell atlas suggests that a stromal-neutrophil axis regulates tissue immunity (6). Metcalfe et al. have reviewed the role of polarized phagocytes in the oral mucosa, suggesting a particular heterogeneity of neutrophils in this tissue. The role of neutrophils in the pathology of the periodontal pocket was recently reviewed (13, 14) and up-regulation of neutrophil activation in inflammatory disease with oral manifestations has been described (15). Ozuna et al. have demonstrated that gram-negative bacteria associated with periodontal disease are able to *highjack* neutrophil function by upregulating azurophilic granule exocytosis as survival mechanism. The plasticity of neutrophils in oral tissues is highlighted suggesting that these cells are underestimated (16). The use of the neutrophil/lymphocyte ratio was suggested as an indicator of disease progression (17).

Zhang et al. have reviewed the role of T-regulatory cells in the context of Head and Neck cancers as well as periodontitis. Other studies suggest that a uniquely large population of FOXp3^+^ Tregs are found in the oral mucosa (18). Tregs promote the generation of IL-17-producing Th17 cells by consuming IL-2, an important survival factor, but also a negative regulator of Th17 differentiation (19).

IL-17 is an important cytokine in the oral mucosa and has been shown to regulate host-microbe interaction (20) as well as being up-regulated in response to mastication (5). Its role in the pathology of periodontitis is well documented (21). In contrast some Th17 cells act as negative regulators of inflammation by secreting IL-10 and are thus regarded as non-pathogenic (22). In this context oral mucositis was shown to be mitigated by IL-17 receptor signalling in a clinically relevant murine model of irradiation induced mucositis which might lead to therapeutic interventions (Saul-McBeth et al.)

The use of pro-biotics is a topic of popular interest for restoring/maintaining the *normal* microbiome (23, 24). Wang et al. suggest this might be a useful adjunct in oral cancer therapy as demonstrated in a mouse model of mucositis using *Strep. salivarius* K12 to help reconstitute the dysbiotic oral microbiome.

Other materials have been suggested as potential down regulators of inflammation and Mooney et al. present a proof-of-concept study using Quercetin, a plant based polyphenolic flavonoid, to modify the oral microbiome both *in vivo* and *in vitro*. This flavonoid has been shown to have health benefits in humans and animals (25) and works through modulation of the NF-кB:A20 axis.

Molecular profiling of multiple cell types and multiple effector molecules provides large amounts of data and moves analysis of health and disease from reductionist theory to systems biology. Profiles of multiple pro-resolving lipid mediators have been investigated by Lee et al. in periodontal inflammation, suggesting a correlation between these molecules, receptor genes and the sub-gingival microbiome that have the potential to skew the periodontal microbiome. Failure to resolve inflammation was investigated by Alvarez et al. in experimental periodontitis where the T cell component was inhibited, but neutrophil and macrophage infiltration was unaffected by early administration of RvE1. Previous studies have suggested that salivary levels of lipid mediators might be used as indicators of health and disease of the periodontium (26)

Salivary flow in the oral cavity is greater than 1 liter/day and the surface epithelium is constantly in turnover. About 70% of the total numbers of lymphocytes are found in the gut (27) and the compartmentalization of the mucosal immune system has been investigated, particularly in the context of vaccination (28–30). While the systemic and mucosal immune systems are regarded as distinct there is constant communication between them in order to maintain the homeostasis of “health”. Many systemic diseases present with oral manifestations and oral health has a considerable impact on systemic disease (31).

Classically IgA has been regarded as the predominant functional antibody in saliva and during the SARS-CoV-2(Covid) pandemic it has been surprising that this antibody has been somewhat neglected (32). Most analysis of immune responses have concentrated on the serum IgG response to this mucosal infection.

Chellamuthu et al. present data on IgG antibodies in the saliva of SARS-CoV-2 infected individuals and suggest this would be an effective alternative to serum-based assays. Secretory IgA (sIgA) antibodies in individuals that have ***not*** been infected with SARS-CoV-2 have been reported (33). However, these cross-reactive antibodies may be a result of the ability of sIgA to bind virus in a non- specific manner as part of its function of virus exclusion from mucosal surfaces. While the contribution of IgA to the immune response to SARS-CoV-2 requires further investigation, a recent review elaborated a greater degree of interaction between IgG and IgA (as well as IgM and IgD) in driving sIgA responses in increasingly complex cross-talk between the mucosal and systemic systems (34).

The detection of soluble mediators of inflammation (cytokines) in serum compared with saliva was highlighted by Novak et al. Salivary cytokine profiling would be less invasive than blood sampling and might reveal more accurately, either disease activity or disease progression in individuals with oral manifestations of disease, and host and environmental influences (35).

Immunohistochemical signatures as clinical monitoring for mucosal melanoma are presented by Xu et al. This disease is difficult to diagnose with a poor prognosis and methods that improve outcomes are very important (36).

Similarly, genetic markers can provide indicators of progression, manifestations and the propensity for diseases. A case report of a family with hereditary gingival fibromatosis (HGF) presented by Gao et al. suggest that the presence of high levels of Human β defensins in the gingival tissues had a beneficial effect in preventing the thickening of the gingiva in this condition.

Qian et al. have used transcriptomic analysis to identify a new set of inflammation promoting cell subsets in chronic periodontitis. Their study shows NLRP3^+^ macrophage involvement and expression of HLA-DR on endothelial cells and CXCL13^+^ fibroblasts were highly associated with regulatory profiles in Asian patients. This is consistent with recent papers on the involvement of NLRP3^+^ cells in periodontal disease (37) and in periodontitis associated with uncontrolled type-2 diabetes (38).

A recurring theme in several submissions is the diversity and plasticity of the cells of the oral mucosa. This may reflect the observations that the oral mucosa, like fetal tissues heals quickly without scarring. The immaturity and diversity of some cell types within the oral mucosa may be the key to the unique physiology of this tissue.

Future studies specifically designed to further develop the oral mucosa and saliva as analytical tools for both oral and systemic disease are an exciting prospect both in the context of new technologies such as multi-colored flow cytometry, multiplex analysis and fast affordable genetic analysis and as potential routes for vaccinology and drug delivery (39).

## Author Contributions

LB drafted the editorial and ND, PS and HK contributed to the final submitted version. All authors contributed to the article and approved the submitted version.

## Conflict of Interest

Author HK was employed by company Intravacc.

The remaining authors declare that the research was conducted in the absence of any commercial or financial relationships that could be construed as a potential conflict of interest.

## Publisher’s Note

All claims expressed in this article are solely those of the authors and do not necessarily represent those of their affiliated organizations, or those of the publisher, the editors and the reviewers. Any product that may be evaluated in this article, or claim that may be made by its manufacturer, is not guaranteed or endorsed by the publisher.
